# Photosynthetic product allocations of *Pinus massoniana* seedlings inoculated with ectomycorrhizal fungi along a nitrogen addition gradient

**DOI:** 10.3389/fpls.2022.948676

**Published:** 2022-08-12

**Authors:** Sun Pengfei, Shen Yafei, Wang Lijun, Chen Tian, Zhang Meng, Xiao Wenfa, Cheng Ruimei

**Affiliations:** ^1^Chinese Academy of Forestry, Key Laboratory of Forest Ecology and Environment of National Forestry and Grassland Administration, Ecology and Nature Conservation Institute, Beijing, China; ^2^Co-innovation Center for Sustainable Forestry in Southern China, Nanjing Forestry University, Nanjing, China

**Keywords:** nitrogen addition, ectomycorrhizal fungi, *Pinus massoniana*, photosynthetic products, ^13^C allocation

## Abstract

Quantifying the allocation of photosynthetic products among different carbon (C) pools is critical for understanding and predicting plant C turnover response to climate change. A field experiment with ectomycorrhizal fungi (EMF) and nitrogen (N) was established to investigate the effects on allocation of photosynthetic products in *Pinus massoniana* (Lamb.) seedlings given increased N deposition. Seedlings were subjected to N addition and symbiosis with EMF, and the short-term allocation of a ^13^C photosynthetic pulse into leaves, branches, stems, roots, and soil was traced. Photosynthetic rate and root respiration were measured. It was found that N addition changed the allocation pattern of photosynthetic products in various organs of *P. massoniana.* Furthermore, N addition, mycorrhizal symbiosis, and interaction of N and EMF, all increased the amount of C produced by photosynthesis. N application less than 60 kg N hm^–1^ a^–1^ could promote the transfer and allocation of photosynthetic products in *P. massoniana* organs, which peaks at 60 kg N hm^–1^ a^–1^, and the highest N treatment began to decrease at 90 kg N hm^–1^ a^–1^. EMF inoculation could expand the absorption area of plant roots to obtain more nutrients and synthesize more C and N compounds for promoting the growth of itself and the host plant, improving the net photosynthetic rate and the distribution of C produced by photosynthesis in various organs. This forms a benign C and N cycle, thereby reducing the effect of high N addition on plants. The optimal N addition concentration was 60 kg N hm^–1^ a^–1^, and the optimal EMF was Pt, which provides a theoretical basis for inoculating EMF during increasing N deposition in the future climate change scenario. This enables plants to distribute more photosynthetic products to their roots, thus affecting their own C distribution for promoting growth.

## Introduction

With the excessive emission of active nitrogen (N) compounds produced by anthropogenic activities, atmospheric N deposition is increasing, and its impact on ecosystem has become a hot topic in global environmental quality and climate change (Fowler et al., 2013; Fu et al., 2020). As the largest developing country, China is one of the three hot spots of high N deposition worldwide. Although the rapid growth of N deposition has stabilized, the total amount remains at a relatively high level (Yu et al., 2019). Atmospheric N deposition interferes with the carbon (C) cycle and ecosystem C accumulation by affecting plant growth, C sequestration, and photosynthetic product allocation (Fan et al., 2011). The translocation and allocation of photosynthetic products determine plant growth and whole-plant biomass allocation (Ho, 1988; Geiger et al., 1996). The translocation of photosynthetic products from source leaves was driven by the hydrostatic gradient between the source and sink (Münch, 1932). However, excessive N will lead to nutritional imbalance in trees, inhibit net photosynthetic capacity, and restrict plant growth (Liu et al., 2011). At present, the effects of N deposition on ecosystems or individual trees are mainly studied through artificial N addition, but the response process of the allocation of photosynthetic products to N addition is different. *Populus tomentosa* seedlings were experiencing C limitation under N addition, and plants regulated photosynthesis by changing leaf thickness for increasing their C sequestration capacity (Gao et al., 2021). With N addition, *Schima superba* and *Cryptocarya concinna* decreased the allocation proportion of photosynthetic products in root, increased the allocation of leaf and shoot, and decreased the ratio of root to shoot (Li et al., 2004, 2005). *Quercus* spp. transport more photosynthetic products to stem and roots (Wang et al., 2016). The root biomass of *Cunninghamia lanceolate* increased significantly under continuous high N addition (Ji et al., 2020).

Ectomycorrhizal fungi (EMF) can symbion with a large number of plants. When plants experience mycorrhizal association, their young roots are closely wrapped by fungal mycelium for growing hyphal sheaths, which can continue to grow mycorrhizal hypha instead of root hairs and produce extensive extraradical mycelia to the soil (Teramoto et al., 2016). Organic and inorganic N compounds in the soil are absorbed by EMF, which through an extensive network of external root hypha, absorb N compounds and transport them to the host plant in exchange for C compounds (Stuart and Plett, 2020; Mayerhofer et al., 2021). Some researchers used ^13^C pulse labeling of plants to study the allocation of photosynthetic products in mycorrhizal symbiosis. They found that the host plant allocated 10–30% of photosynthetic products to symbiotic root system (Eissenstat et al., 2015). In the experiment of *Pinus muricata* seedlings inoculated with EMF, it was found that plants preferred to allocate the nearest photosynthetic products to the mycorrhizal roots where more N sources could be obtained (Bogar et al., 2019). More ^14^C compounds produced by photosynthesis were absorbed from the roots of l *Pinus densiflora* seedling inoculated with EMF, but the ^14^C compounds consumed by soil respiration were the same as those absorbed by mycorrhiza (Wu et al., 2002). The ^13^C compounds produced by photosynthesis in Douglas fir (*Pseudotsuga menziesii*) with EMF inoculated into the root increased significantly (Pickles et al., 2017).

China has the largest plantation in the world. Afforestation is crucial for regulating climate, reducing CO_2_ emissions (Meng et al., 2018), and achieving the goal of C peak by 2030 and C neutralization by 2060 in China (Wang et al., 2009). *Pinus massoniana* (Lamb.), which is a pioneer tree species of afforestation in Southern China, is also a typical ectomycorrhizal tree species. The research on the symbiotic relationship between *P. massoniana* and ectomycorrhiza has been conducted for a long time. As early as 1989, Chen investigated and identified the symbiotic mycorrhiza of *P. massoniana* and found that a total of 27 species of EMF could symbiosis with it (Chen, 1989). Among them, *Suillus grevillei* (Sg) and *Pisolithus tinctorius* (Pt) are typically excellent symbiotic agents of *P. massoniana* (Chen and Pei, 1989). The inoculation experiment of five kinds of EMF in *P. massoniana* seedlings showed that EMF could promote the height and diameter of seedlings and improve their photosynthetic capacity (Wang and Tu, 2021). Under drought and shade, its photosynthetic products were limited, and plants would increase the allocation ratio in roots and reduce the release rate of recent photosynthetic products from soil respiration for environmental adaptation (Deng et al., 2021).

To better understand the EMF response to photosynthetic product allocation under different N addition quantities, and to elucidate whether EMF alleviates the negative effects of high N deposition on plants, clarification on (i) whether inoculation with EMF can increase the distribution of C in plants with recent photosynthesis, and (ii) whether seedling growth would increase with N additions, was necessary. We anticipated a possible dysfunctional response due to over fertilization at high N addition levels, but EMF can alleviate this negative phenomenon. To address these questions, a pulse-chase study of ^13^C allocation to leaves, branches, shoots, roots, and soil was conducted over 30 days to quantify the short-term patterns of response of C allocation to the N and EMF treatment.

## Materials and methods

### Research site

**FIGURE 1:**
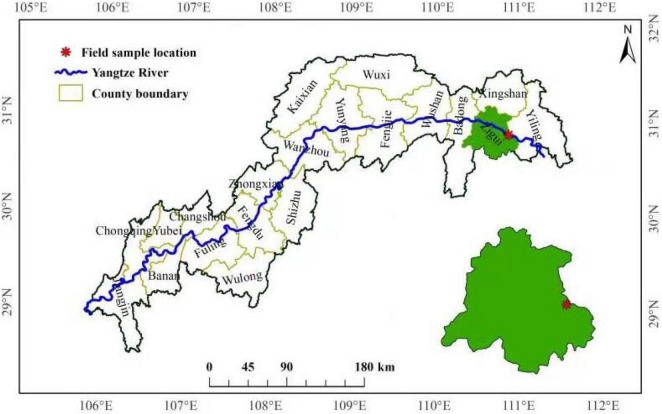
Diagram of the field experimental site.

Experiments were conducted at the Forest Ecosystem State Positioning Observation Station in the Three Gorges Reservoir area (110°54′E, 30°53′N, 375 m altitude), Zigui County, Hubei Province, China (Figure 1). *P. massoniana* is one of the main coniferous species in this area, and the proportion of total area and total stock is as high as 48.8 and 64.2% (Ge et al., 2017), and the N deposition is 30 kg N hm^–1^ a^–1^ (Ding et al., 2020).

### Ectomycorrhizal fungi and plant materials

We designed a two-factor randomized block treatment. The first factor was EMF, which were Sg, Pt, and control (CK), respectively, EMF were provided by the Institute of Forest Ecological Environment and Nature Conservation, Chinese Academy of Forestry Sciences. The second factor was N addition treatment, which was divided into four levels according to the annual atmospheric N deposition in this area: 0 kg N hm^–1^ a^–1^, 30 kg N hm^–1^ a^–1^ (normal deposition), 60 kg N hm^–1^ a^–1^ (moderate deposition), and 90 kg N hm^–1^ a^–1^ (excessive deposition). There were a total of 12 treatments, and each treatment had 100 pots, with a total of 1,200 pots. Before the N addition, the seedlings grew for 3 months to ensure the symbiotic relationship between seedlings and EMF. After that, N addition was conducted once a month, and the NH_4_NO_3_ solution of 0, 0.714, 1.428, and 2.143 g/L was added each time, respectively.

In March 2021, 1,200 one-year-old *P. massoniana* seedlings were randomly assigned to greenhouse, and each was planted in a separate pot (20 cm in diameter and 15 cm in height), and filled with 3 kg of soil. The soil was collected from the *P. massoniana* forest stands within 2 km of the experimental site and stand by after high-temperature sterilization. The basic physical and chemical properties of the experimental soil were as follows: total soil N was 1.01 g kg^–1^, total soil P was 0.54 g kg^–1^, total soil K was 1.53 g kg^–1^, available soil N was 47.28 mg kg^–1^, available soil P was 8.97 mg kg^–1^, available soil K 90.17 mg kg^–1^, organic matter content was 13.97 g kg^–1^, and pH was 5.97.

### ^13^C-Labeling experiments

**FIGURE 2:**
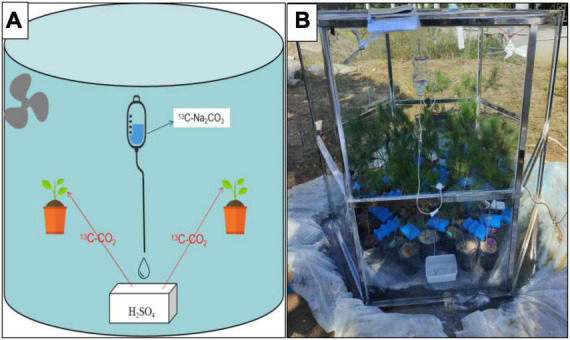
**(A)** Schematic drawing of the setup. ^13^C-Na_2_CO_3_ was dripped into H_2_SO_4_ solution box through the infusion tube to produce ^13^CO_2_, which was then absorbed by plants. **(B)** Photo of a plant used in the labeling experiment.

The ^13^C–CO_2_-labeling took place under natural daylight conditions (cloudy, partly sunny) on 12th October 2021. In total, 180 seedlings were randomly selected for each treatment, labeled with ^13^CO_2_ in the morning (7:00–11:00). The ^13^CO_2_ pulse labeling was conducted in a homemade Plexiglas chamber. To avoid gas leakage, the chamber was placed in a concave groove and sealed with water. The tiny hole left by the injection was covered with scotch tape to avoid gas leakage. To guarantee a uniform distribution of ^13^CO_2_, two fans were installed inside the chamber and were used to mix the air thoroughly during the labeling.^13^CO_2_ was produced by 1 N ^13^C–Na_2_CO_3_ solution and 1 N H_2_SO_4_ solution. Using an LI-8100 CO_2_ infrared gas analyzer (LI-COR, Inc., American) to continuously monitor the concentration of carbon dioxide in the chamber. When the initial CO_2_ levels had declined to 100 μl L^–1^, H_2_SO_4_ was added to the vial to increase the CO_2_ concentration to 400 μl L^–1^. Once all of the ^13^C–Na_2_CO_3_ was used and the total CO_2_ concentration decreased below 400 μl L^–1^, H_2_SO_4_ was added to the flask with Na_2_CO_3_ to maintain the CO_2_ concentration to 400 μl L^–1^. This process was repeated approximately 8 times before the Plexiglas chamber was removed (Figure 2).

### Plant tissue and soil sample collection and analysis

Leaves, branches, shoot, root, and soil samples were collected on the labeling day before (coded as 0 day) and the following 1, 5, 10, 21, and 30 days after labeling. Plant aboveground parts of all species were harvested and pooled as shoot samples by clipping at the soil surface. Soil cores (5 cm in diameter) were taken to 15-cm depth. All roots and soil in the cores were carefully extracted and sieved with a 2-mm sieve. Soil samples passed through the sieve were air-dried for total C and ^13^C analysis. The sampled roots were carefully washed by wet sieving through a 0.5-mm sieve to remove attached soil and debris. Shoot and root samples were oven-dried at 65°C for 48 h. The δ^13^C and C contents were determined using an isotope ratio mass spectrometer (Delta V Advantage) coupled with an elemental analyzer (Flash 2000 EA-HT) (Thermo Fisher Scientific Inc., Waltham, MA, United States).

NaOH was used to absorb CO_2_ produced by soil respiration in a certain period of time (Deng et al., 2019). At the end of labeling, a closed breathing chamber containing NaOH solution (1 mol L^–1^) (7.5 cm in diameter and 9 cm in height) was inserted into the basin soil to absorb CO_2_ produced by soil respiration on the 1st, 4th, 5th, 11th, and 9th day after labeling, and the amount of CO_2_ uptake was determined by HCl (1 mol L^–1^).

### Photosynthetic rate measurements

Photosynthetic rates were measured from 9:00 to 11:00 am before collected plants. The top-positioned leaves from one pot were used to establish photosynthetic rates using a portable closed gas-exchange system (LI-6400; Li-Cor, Lincoln). Thirteen light conditions (2,000, 1,600, 1,200, 1,000, 800, 400, 200, 160, 120, 80, 40, 20, and 0 μmo m^–2^ s^–1^) of PPFD were provided using a red–blue LED light source. The concentrations of CO_2_ in ambient air entering the leaf chamber and leaf-to-air vapor pressure deficit were maintained at 350 μl L^–1^ and 1.1 kPa, respectively, during the measurement period.

### Carbon isotope analysis

All C isotope values were reported using the isotope delta values (δ^13^C), that is, ^13^C/^12^C, were expressed in per mil (‰) in relation to the international reference standard:


δC13=[(Rsam-R)std/R]std×1000

where R_sam_ and R_std_ are the ^13^C/^12^C ratios of the sample and standard, respectively.

The atomic percentage of ^13^C in each organ (F*_*i*_*, %), was as follows:


$${\text{F}}_{i}=[({\text{δ}}^{\text{13}}\text{C}+\text{1}000)\times {\text{R}}_{\text{std}}]\times \text{1}00/[({\text{δ}}^{\text{13}}\text{C}+\text{1}000)\times {\text{R}}_{\text{std}}+\text{1}]$$

The total amount of C in each organ (C*_*i*_*, g) was determined as follows:


$$C_{i}=T_{i}\times W_{i}$$

where W_*i*_ is the biomass in each organ, and T_*i*_ is the percentage of C in each organ.

The total amount of fixed ^13^C in each organ (^13^C*_*i*_*, g) was determined as follows:


Ci13=Ci×(Fi-Fn)/100×1000

where F_*n*_ is the ^13^C atomic percentage in the unlabeled sample.

The ^13^C allocation ratio in each organ at each sampling time (P*_*i*_*, %) was as follows:


Pi=Ci13/CF13

where ^13^C*_*F*_* is the sum of the ^13^C in the whole plant.

The ^13^CO_2_ respiration rate in soil (VCO_2_ (soil), mg plant^–1^ day^–1^) was as follows:


VCO(soil)2=mCO2×BA×Δt

where mCO_2_ is the amount of CO_2_ absorbed by NaOH, A is the area of the breathing chamber, B is the area of the mouth of the pot, and Δt is the time to absorb CO_2_.

The variation of the partitioning of the A^13^C_*t*_ (%) in each organ of the plant under different treatment after pulse labeling is given by:


A13Ct=C13tC13total, 1×100

where ^13^C_*t*_ is the total amount of ^13^C in different parts of plants, *t* is different parts, and ^13^C_*total,1*_ is the total ^13^C reserved 1 day after pulse lebeling.

Soil retention and plant respiration [A^13^C_*other*_(mg)] is given by:


$${\text{A}}^{\text{13}}{\text{C}}_{\text{other}}=\text{1}00-{\text{A}}^{\text{13}}{\text{C}}_{\text{plant}}-{\text{A}}^{\text{13}}{\text{C}}_{\text{soilrespiration}}$$

### Data analysis

Statistical analysis was performed using the SPSS 16.0 software package (SPSS, Chicago, IL, United States). A two-factor analysis of variance (ANOVA) followed by Duncan’s multiple comparisons was used. Data shown are the mean standard error (SE). Two-way ANOVA was used to test the effects of the different labeling times and sampling times on the variables under evaluation. All figures were drawn using Origin software 2021 (Origin Lab Corp., Northampton, MA, United States).

## Results

### Differences in the net photosynthetic rate at different time points

The mean value of the net photosynthetic rate at different periods before collecting plants was calculated. Across the N-gradient fertilization, we observed that the net photosynthetic rate of *P. massoniana* inoculated with EMF was higher than CK, it increased steadily with N addition to a peak at N60, and declined at the highest levels. The net photosynthetic rate of *P. massoniana* inoculated with Sg was better than Pt at N0 and N30. In contrast, Pt was better than Sg at N60 and N90. This indicates that the net photosynthetic rate of *P. massoniana* inoculated with EMF was better than that of non-inoculated and was also affected by the concentration of N addition (Table 1).

**TABLE 1:** Comparison of net photosynthetic rate of *Pinus massoniana* in different periods of time.

Inoculation	N treatment (kg⋅N hm^–^^2^ a^–^^1^)	Time after labeling				
		12 October	16 October	21 October	1 November	10 November
Sg	0	8.03	8.15	8.26	7.89	7.92
	30	10.07	10.63	9.58	10.09	9.89
	60	11.15	12.39	12.17	11.97	10.89
	90	11.20	11.92	11.29	11.21	10.57
Pt	0	7.58	8.01	7.72	7.58	7.36
	30	8.43	8.84	8.52	8.66	8.95
	60	12.06	13.22	14.35	13.28	12.89
	90	11.85	12.96	13.20	12.84	12.55
CK	0	7.12	7.53	7.15	6.88	6.94
	30	8.43	7.81	8.59	8.16	8.25
	60	10.44	12.76	10.28	9.54	9.71
	90	9.27	9.79	9.51	8.67	8.86

CK, control. Sg, *Suillus grevillei*. Pt, *Pisolithus tinctorius*. N0: 0 kg N hm^–1^ a^–1^. N30: normal deposition 30 kg N hm^–1^ a^–1^. N60: moderate deposition 60 kg N hm^–1^ a^–1^. N90: severe deposition 90 kg N hm^–1^ a^–1^.

### Short-term dynamics of δ^13^C in different organs

**FIGURE 3:**
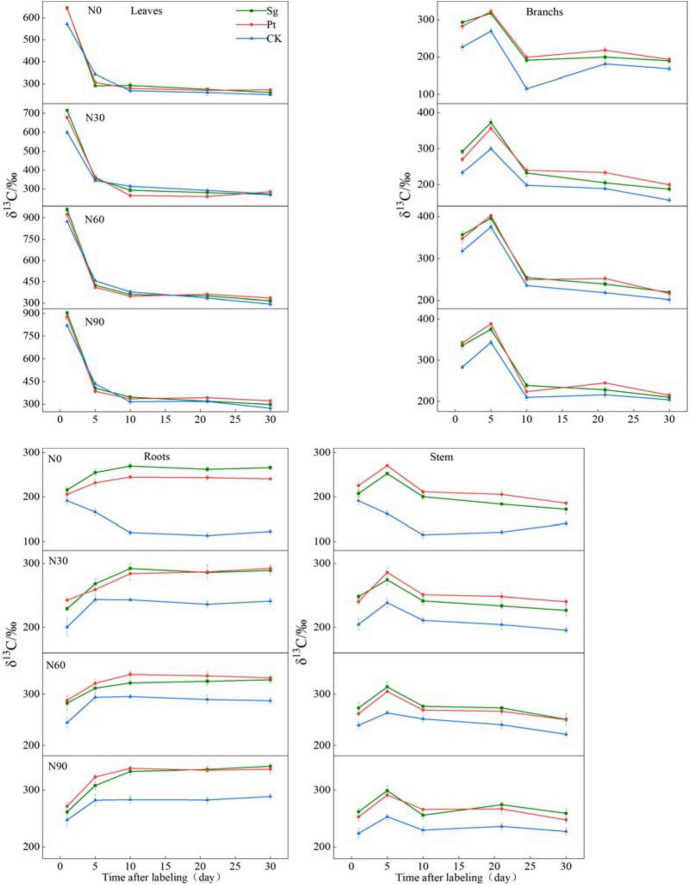
The line chart shows the changes of δ^13^C value in each organ of the plant under different treatment after pulse labeling, and for figures and significant analysis, see Supplementary Tables 1–4. Vertical bars illustrate standard errors of means (*n* = 3). CK: control. Sg: *Suillus grevillei*. Pt: *Pisolithus tinctorius*. N0: 0 kg N hm^–1^ a^–1^; N30: normal deposition 30 kg N hm^–1^ a^–1^; N60: moderate deposition 60 kg N hm^–1^ a^–1^; N90: severe deposition 90 kg N hm^–1^ a^–1^. Date is time after labeling.

The variation trend of δ^13^C in different organs of *P. massoniana* under different treatments was as follows: the δ^13^C sharply declined in leaves after the end of pulse labeling and decreased slowly until reaching the peak in roots, stem, and branches on day 5. During 1–30 days after pulse labeling, the value of the δ^13^C in leaves inoculated with Sg, Pt, and CK decreased by 383.23, 368.76, and 319.70‰, respectively, under N0 (Supplementary Table 1). With increasing N concentration, the δ^13^C in leaves initially increased before decreasing and reached the highest value at N60. The δ^13^C produced by photosynthesis in the leaves completed the transport from the leaves to roots, stem, and branches 1–5 days after the end of labeling. N addition, mycorrhizal symbiosis, and interaction of N and EMF could all increase the output of photosynthetic products from the source leaves during the period of pulse labeling, and the output was the highest at Sg + N60 (Figure 3).

On the first day after pulse labeling, the δ^13^C in roots, stem, and branches was treated with N addition, mycorrhizal symbiosis, and interaction of N and EMF and was higher than those of the CK, indicating that the treatment could promote the transport of photosynthetic products from source to sink during the marking period. The variation trend of δ^13^C in root was gradually increased after the end of labeling, and the value of EMF reached the maximum on the tenth day (Pt + N60) (Supplementary Table 2). During the 1st–5th days, the N treatment value increased before stabilizing, whereas the CK treatment decreased gradually; the minimum value appeared on the tenth day. In the branches and stems, the δ^13^C of all treatments reached the maximum on the fifth day, and all reached the maximum value under N60 treatment, except for the CK (Supplementary Tables 3, 4). The δ^13^C reached the maximum value on the fifth day, then decreased sharply, before stabilizing (Figure 3).

### Soil ^13^CO_2_ efflux rate and accumulated ^13^CO_2_

**FIGURE 4:**
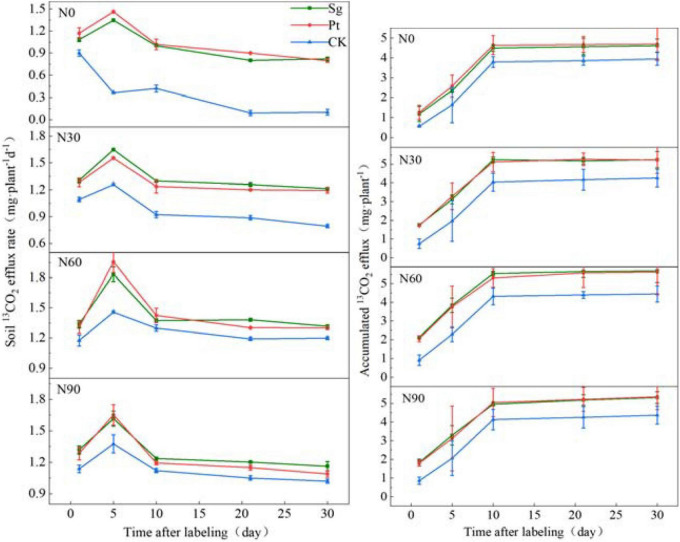
The variation of the soil ^13^CO_2_ efflux rate and accumulation under different treatment after pulse labeling. Vertical bars illustrate standard errors of means (*n* = 3). CK: control. Sg: *Suillus grevillei*; Pt: *Pisolithus tinctorius*; N0: 0 kg N hm^–1^ a^–1^; N30: normal deposition 30 kg N hm^–1^ a^–1^; N60: moderate deposition 60 kg N hm^–1^ a^–1^; N90: severe deposition 90 kg N hm^–1^ a^–1^. Date is time after labeling.

Nitrogen addition, mycorrhizal symbiosis, and interaction of N and EMF increased the rate of ^13^CO_2_ release from soil respiration and delayed the occurrence of the maximum value. The specific performance was as follows: the CK treatment reached the maximum value on the first day after the end of labeling, whereas under N addition, mycorrhizal symbiosis, and interaction of N and EMF, it reached the maximum value on the fifth day after the end of labeling. After reaching the maximum value, the ^13^CO_2_ release rate of soil respiration decreased gradually and then stabilized (Supplementary Table 5). Under all of the N addition concentrations, the soil respiration release rate of mycorrhizal plants was significantly higher than that of nonmycorrhizal plants. The amount of ^13^CO_2_ accumulated in soil increased sharply within 10 days after labeling and then stabilized. It can be seen that the fixed ^13^C was exported within 0–10 days. During this period, the amount of ^13^CO_2_ accumulated in soil inoculated with EMF was higher than that of uninoculated plants at the same level of N addition (Figure 4 and Supplementary Table 6).

### Pulse ^13^C in different carbon pools

**FIGURE 5:**
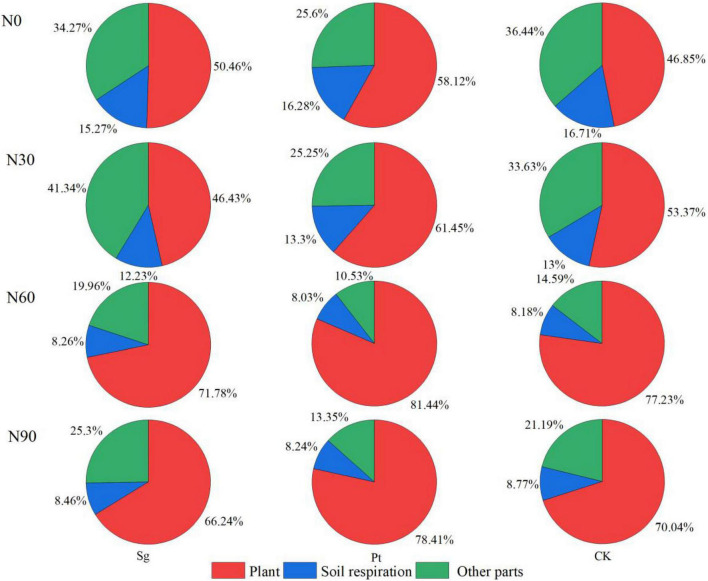
Pie chart shows the weight mean of the ^13^C in different carbon pools under different treatment 30 day after pulse labeling. CK: control; Sg: *Suillus grevillei*; Pt: *Pisolithus tinctorius*; N0: 0 kg N hm^–1^ a^–1^; N30: normal deposition 30 kg N hm^–1^ a^–1^; N60: moderate deposition 60 kg N hm^–1^ a^–1^; N90: severe deposition 90 kg N hm^–1^ a^–1^. Date is time after labeling.

During the labeling period, ^13^C produced by photosynthesis of *Pinus* was finally distributed in three parts: plant, soil respiration, and other parts. On the 30th day after the end of labeling, ^13^C remaining in CK-treated plants accounted for 46.85% of the total fixed amount during the labeling period, 16.71% of ^13^C consumed through soil respiration, and 36.44% of ^13^C in other parts. At the same level of N application, the retention of ^13^C in mycorrhizal seedlings increased, and Pt was higher than Sg. The two kinds of EMF were the largest at N60, and Sg and Pt accounted for 71.78 and 81.44%, respectively. Mycorrhizal symbiosis also reduced the proportion of ^13^C released from soil respiration. With the increase of N application concentration, the ^13^C retained by plants increased, reached the maximum distribution ratio at N60 and decreased at N90. Compared with CK, when inoculated with Sg and Pt in N60, the retention ratio of ^13^C in plant increased by 24.93 and 34.59%, respectively. The results showed that the N addition, mycorrhizal symbiosis, and interaction of N and EMF increased, whereas N application decreased the recent photosynthate retention ratio in the plant (Figure 5).

### Pulse ^13^C in each organ of the plant

**FIGURE 6:**
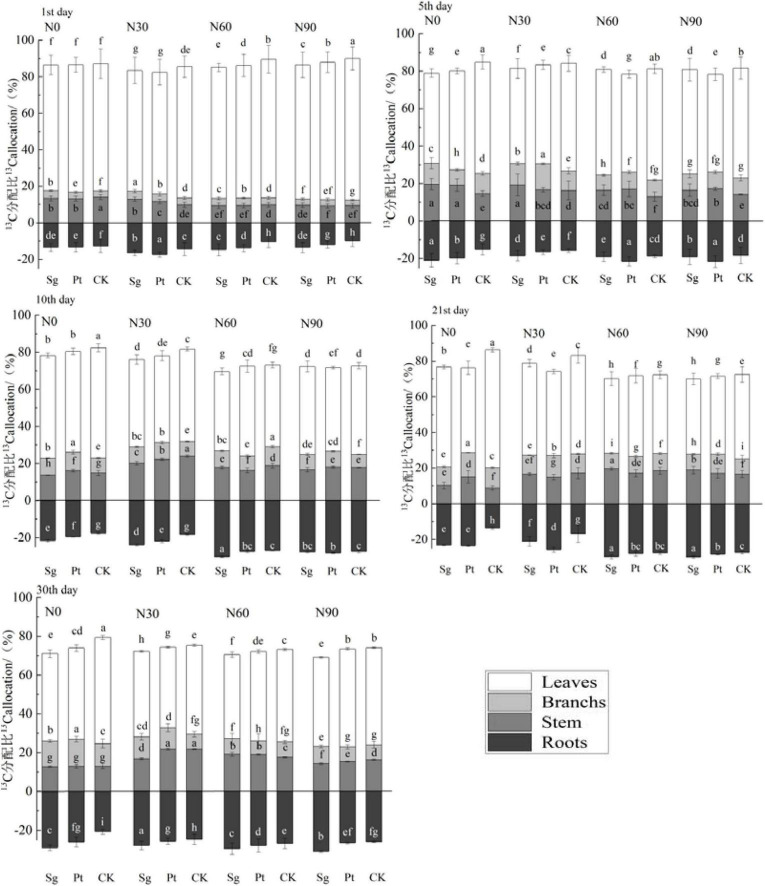
Bars show the variation of the partitioning of the ^13^C in each organ of the plant under different treatment after pulse labeling. Error bars represent the standard error; *n* = 3. Differences in treatments were analyzed with the Kruskal–Wallis rank-sum test. Significant tests (*p* < 0.05) were followed by Duncan’s test of multiple comparisons, and significant differences are indicated by lowercase letters. CK: control; Sg: *Suillus grevillei*; Pt: *Pisolithus tinctorius*; N0: 0 kg N hm^–1^ a^–1^; N30: normal deposition 30 kg N hm^–1^ a^–1^; N60: moderate deposition 60 kg N hm^–1^ a^–1^; N90: severe deposition 90 kg N hm^–1^ a^–1^. Date is time after labeling.

The distribution proportion of ^13^C in leaves decreased with increasing labeling time, whereas increased with the increase of labeling time. EMF increased the output of ^13^C from leaves and decreased the distribution ratio of ^13^C in leaves. On the 30th day after the end of labeling, the distribution ratio of ^13^C inoculated with Sg and Pt in leaves increased by 8.83 and 7.71%, respectively, compared with CK. The distribution ratio of ^13^C in roots inoculated with Sg and Pt increased by 7.62 and 4.80% under N0, respectively. Within 1–10 days after the completion of ^13^C transfer, the increase of ^13^C distribution ratio of ^13^C with EMF was higher than that of the CK under N0 and N30, but lower than that of the CK under N60 and N90 concentrations. However, the ^13^C distribution ratio in roots initially increased and then decreased with N addition. The distribution ratio was highest at N60, indicating that the N application could promote the recent photosynthate consumption of *Pinus* roots (Figure 6).

## Discussion

### Ectomycorrhizal fungi promote the photosynthetic products transfer to roots

The transport of C compounds produced by photosynthesis is affected by EMF and environmental N concentration. Högberg traced ^13^C compounds produced by photosynthesis in pine forests and found that ^13^C compounds were quickly transferred to mycorrhizal mycelium and redistributed, but excessive N application significantly reduced their distribution to the underground part of plant (Högberg et al., 2010). In our study, under the treatment of EMF, δ^13^C in leaves decreased sharply after labeling and greatly slowed down on the 5th day. δ^13^C in stems and branches increased to the highest value on the 5th day after labeling, whereas δ^13^C in roots continued toward the peak on the 10th day, and δ^13^C was significantly higher than that of the CK. Under the concentration of N0 and N30, the effect of Sg inoculation was better than that of Pt. Contrarily, the effect of Pt inoculation was better than that of Sg under N60 and N90, indicating that the transport rate of photosynthate from source leaf to root was affected by EMF. Inoculation with EMF delayed the process when δ^13^C peaked in roots, which was consistent with the results of Högberg et al. (2010) and Leake et al. (2001). It is well-known that since EMF can form a good symbiotic relationship with the host plant, it can promote the growth of itself and host plants by expanding the root absorption area for obtaining more nutrients (Kayama and Yamanaka, 2014; Zhang et al., 2017). When the growth of trees was limited due to low soil nutrient content, EMF could help trees obtain growth-limiting nutrients, such as C, N, and P, through more mycelia formed by symbiosis with tree roots. This improves the photosynthetic C assimilation ability of host plants (Liu, 2017; Plett et al., 2020).

In the ^14^C labeling experiment of *Pinus sylvestris var. mongolica*, it was found that ^14^C photosynthetic products were quickly transported to the root within 1 day after the end of labeling and transferred to the external mycelium within 3 days. The higher the mycelium density, the higher the ^14^C photosynthate (Wu et al., 2001). EMF was a strong C sink, and sufficient nutrients could increase the amount of photosynthetic products and the distribution of photosynthate from source to sink of *Pinus*, whereas the transport of C compounds from root to aboveground was conducted through the soil outside the hypha (Simard et al., 1997; Gorka et al., 2019). In our study, both kinds of EMF could promote the amount of δ^13^C in various organs of *Pinus*, which was significantly higher than that of the CK. However, there was a difference between the two kinds of EMF, which may be due to the difference in the number of mycelium formed by the young root and the number of mycelium that continued to grow after the symbiosis of different EMF and host plants. This resulted in differences in nutrient absorption capacity due to different root richness. EMF delays the time when δ^13^C reaches the peak value in roots, which may be because photosynthetic products can promote the extension of hypha. These are used by EMF for its own growth and extension, and δ^13^C can be transferred between mycelium. It is seldom transported to the shoot.

The availability of soil N influences the morphological characteristics of soil. It has been found that high N supply promotes plant root development and leads to larger root biomass (Kraiser et al., 2011; Meng et al., 2016). In our study, δ^13^C in roots, stems, branches, and leaves of *P. massoniana* initially increased before decreasing with increasing concentration of N application. All ratios reached the peak on the 5th day after the end of labeling, reaching the highest at N60 and declined at N90, which was consistent with the results of *Pinus tabulaeformis* (Wang et al., 2013) and *poplar* (Meng et al., 2018). The results showed that in the range of medium concentration N deposition, N addition promoted the photosynthesis of *Pinus* and increased the transport of δ^13^C to the sink organs, which was mainly attributed to the fact that when N was sufficient, the source leaves transported enough photosynthates to the sink tissue to ensure the growth of roots, so that the roots synthesized C and N compounds beneficial to plant growth and formed a benign C and N cycle (Schlüter et al., 2012). When the concentration of N application exceeded this range, it began to decrease, and the effect could be weakened when the N concentration was excessive after inoculation with EMF.

### Effects of N and ectomycorrhizal fungi on the soil ^13^CO_2_ efflux rate and accumulation

Pulse labeling experiment had become a common method to study assimilation, distribution, and respiration in physiology and ecology (Bowling et al., 2008). Epron et al. (1999) found that when photosynthetic products were synthesized, the release from soil respiration was mainly concentrated in the early stage after photosynthate synthesis. In our study, during 1 to 10 days after labeling, the release of ^13^C from photosynthesis in soil respiration increased sharply, before stabilizing, indicating that the ^13^C products consumed by soil respiration were mainly the recent products of plant photosynthesis. N addition, mycorrhizal symbiosis, and interaction of N and EMF all increased the total release of photosynthates from soil respiration. At the same the N application concentration, the ^13^C produced by soil respiration of *P. massoniana* inoculated with EMF was higher than that of uninoculated plants, but with increasing N concentration, the ^13^C produced by soil respiration initially increased before decreasing, with the highest at middle N concentration (N60), and decreased at the highest N application concentration (N90). The results showed that the C emission from soil increased with the increase of photosynthate synthesis, and N addition and EMF could improve the photosynthetic capacity of plants, thus affecting the process of C cycle in plants.

Nitrogen addition, mycorrhizal symbiosis, and interaction of N and EMF all delayed the time when the rate of soil respiration and release of ^13^CO_2_ reached the peak value, indicating that treatments could enhance the recent release of photosynthetic products from soil respiration in *P. massoniana.* Soil respiration was highest at medium N concentration (N60) (Pt > Sg), which may be because the photosynthetic C input in the underground was closely related to the growth rate of roots. Suitable N concentration and EMF improved the soil environment of plant roots (Ge et al., 2015). Plants had sufficient nutrition and root activity to transport more C and enhance root respiration during the growing period. Simultaneously, N addition and EMF promoted the ability of photosynthesis of *Pinus* and made plants produce more photosynthates, which also increased the plant consumption of recent photosynthate transformation and respiration. In addition, N addition and EMF inoculation reduced the retention time of recent photosynthates in source leaves, thus increasing the release rate of recent photosynthates from soil respiration. However, the rate of ^13^CO_2_ release from soil respiration began to decrease with excessive N concentration, but in the mycorrhizal plants it was significantly higher than that of nonmycorrhizal plants.

### Ectomycorrhizal fungi promote the content of photosynthetic products in various organs

There are three ways for plants to distribute new photosynthetic products, including absorption and utilization of plant organs, retention of soil organic matter through soil respiration or transformation into soil, and atmospheric release in the form of CO_2_ (Wu et al., 2010). During the whole tracer period, N addition, mycorrhizal symbiosis, and interaction of N and EMF all increased the retention ratio of photosynthate in plants, and the retention ratio of plants inoculated with Pt at N60 was the highest. The results showed that EMF was beneficial to the allocation of photosynthetic products in the plant during N deposition in a certain range. On the 1st to 5th day after labeling, with the increase of N addition, the output rate of ^13^C in leaves inoculated with EMF increased with increasing photosynthesis, which was consistent with the overall increment of root, stem, and branch absorption. This phenomenon was consistent with the C distribution from source leaves to sink organs of *P. tabulaeformis* as observed by Wang and Liu (2014). The amount of C allocated to the root mainly depends on the amount produced by plant photosynthesis (Farrar and Jones, 2000). A certain range of N application and EMF promoted the production of more photosynthetic products in *Pinus*, which increased the photosynthetic products allocated to the root system. The root system had enough nutrients to maintain the growth of itself and the plant (Zong et al., 2018), and the symbiosis of hypha and root increased the absorption and transport area of the plant. This can distribute more C to the deeper soil, whereas excessive N concentration would lead to the imbalance of nutrient elements in the soil, thereby reducing the distribution of photosynthetic products to the root system. Under the suitable N application rate, the ^13^C content in leaves and roots of *Pinus* inoculated with EMF increased significantly, indicating that the C distribution in the roots and leaves with the most active metabolism was controlled by N application rate and EMF “pull” (Farrar and Jones, 2000). When the aboveground C sink was sufficient, the mycorrhiza had enough “pulling force” to distribute ^13^C to each sink organ, and the gradient difference of N concentration led to the difference of C distribution. At the highest N addition (90 kg N hm^–1^ a^–1^), the favorable effect on the allocation of photosynthetic products began to decline, but the negative effect could be attenuated by EMF.

Previous studies have shown that plants can better maintain their growth under adequate nutrient supply and suitable growth environment, increase the biomass of aboveground and belowground parts, and enhance photosynthesis to produce more C compounds and distribute them to various organs (Reynolds and Thornley, 1982; Zong et al., 2018). However, N deficiency increases the distribution of photosynthate to the roots of different plant types, shrubs, herbs, or woody plants (Cronin and Lodge, 2003; Vanninen and Mäkelä, 2005; Grechi et al., 2007). This was because N deficiency reduces the transport of cytokinin from root to stem, reduces the rate of cell division, and reduces the transport of C from phloem to stem, which causes C to accumulate around the phloem, increasing pressure on leaves. Meanwhile, root cells continue to divide, resulting in a pressure difference between source leaf and sink root, and relatively more photosynthetic products are allocated to the root (Ping et al., 2010). But adequate N supply and inoculation of EMF enabled the plant to produce more photosynthetic products, whereas larger root area and biomass also ensured the distribution of photosynthetic products to all parts. In our study, it was found that EMF could promote the distribution of newly fixed photosynthetic products in plants, and inoculation of EMF slowed down the decrease of photosynthetic products caused by high N deposition. Therefore, inoculation of EMF and a certain range of N deposition (less than 60 kg N hm^–1^a^–1^) could promote the allocation of photosynthetic products in *Pinus*.

## Conclusion

Our results show the transport and distribution of photosynthetic products in *P. massoniana* seedlings increased under the influence of N addition and EMF inoculation. N deposition changed the aboveground and belowground allocation pattern of photosynthetic products, and N addition less than 60 kg N hm^–1^a^–1^ could promote the transfer and distribution of photosynthetic products in *Pinus* organs. The amount of N application began to decrease when the amount of N application was 90 kg N hm^–1^ a^–1^. N addition, mycorrhizal symbiosis, and interaction of N and EMF could increase the amount of C produced by photosynthesis. After EMF inoculation, it can promote the transport and allocation of ^13^C to plant roots and stems to promote plant thickening and growth. The optimal N application concentration was 60 kg N hm^–1^a^–1^, and the optimal EMF was Pt. This study provides a theoretical basis for plants to distribute more photosynthetic products to the roots under the increase of N deposition in the future climate change scenarios, thus affecting their own C allocation for promoting growth.

## Data availability statement

The original contributions presented in this study are included in the article/Supplementary material, further inquiries can be directed to the corresponding author.

## Author contributions

SP and CR envisioned and wrote the manuscript. SY, WL, CT, and ZM did the experimental work, which was supervised by XW. All authors contributed to the article and approved the submitted version.
