# Supplementary material for: Photosynthetic product allocations of Pinus massoniana seedlings inoculated with ectomycorrhizal fungi along a nitrogen addition gradient
Source: Front Plant Sci. 2022 Aug 12;13:948676. doi: 10.3389/fpls.2022.948676 (PMC9412729; doi:10.3389/fpls.2022.948676)
Supplement: Supplementary file 1 [file Data_Sheet_1.docx]

**Supplementary Material**

**Table S1** | The changes of δ^13^C value in leaves of the plant under different treatment after pluse-labelling

| N concentration（kg·N·ha^-2^·a^-1^） | Inoculation | 1^st^ days | 5^th^ days | 10^th^ days | 21^st^ days | 30^th^ days |
| --- | --- | --- | --- | --- | --- | --- |
| N0 | Sg | 644.76（5.83）g | 291.56（4.52）g | 293.20（15.50）ef | 275.23（7.00）ef | 261.53（8.47）ef |
|  | Pt | 641.99（7.97）g | 306.87（10.83）g | 279.75（8.53）fg | 270.72（7.16）ef | 273.23（5.47）de |
|  | CK | 570.89（9.53）h | 342.80（5.58）f | 269.22（9.90）g | 260.82（13.59）f | 251.19（6.09）f |
| N30 | Sg | 714.54（4.60）e | 354.91（4.98）ef | 293.45（8.38）ef | 280.66（5.96）de | 270.66（5.71）e |
|  | Pt | 678.08（7.90）f | 364.11（5.29）e | 265.01（11.81）g | 260.28（10.95）f | 285.19（8.62）cd |
|  | CK | 599.79（7.77）h | 344.26（5.81）f | 314.34（7.26）de | 292.39（6.49）d | 273.30（2.94）de |
| N60 | Sg | 957.89（12.31）a | 425.51（6.98）b | 358.86（11.68）ab | 349.56（7.03）ab | 316.24（6.27）b |
|  | Pt | 922.98（23.41）b | 410.34（11.00）bc | 346.85（4.71）abc | 361.18（9.33）a | 334.31（12.09）a |
|  | CK | 873.20（3.23）c | 456.42（6.37）a | 378.18（12.24）a | 334.81（8.85）b | 292.93（11.83）c |
| N90 | Sg | 902.05（13.08）bc | 406.26（6.37）c | 347.16（11.54）abc | 319.74（7.66）c | 298.30（11.11）c |
|  | Pt | 877.24（17.16）c | 384.99（11.28）d | 336.36（19.13）cd | 343.03（9.53）b | 322.24（4.45）ab |
|  | CK | 819.25（8.30）d | 434.09（12.06）ab | 317.06（18.92）d | 317.99（9.86）c | 273.95（15.30）de |

Note: Vertical bars illustrate standard errors of means(n=3). CK: control. Sg: *Suillus grevillei.* Pt: *Pisolithus tinctorius*. N0: 0 kg·N·hm^-1^a^-1^. N30: normal deposition 30 kg·N·hm^-1^a^-1^. N60: moderate deposition 60 kg·N·hm^-1^a^-1^. N90: severe deposition 90 kg·N·hm^-1^a^-1^. 1^st^ -30^th^ days is the date after pluse-labelling. Numbers show the weighted mean of each treatment. Standard error is shown in brackets(p＜0.05). Different letters indicate significant differences (Differences in treatments were analyzed with the Kruskal-Wallis rank sum test. Significant tests (p < 0.05) were followed by Duncan's test of multiple comparisons).

**Table S2** | The changes of δ^13^C value in roots of the plant under different treatment after pluse-labelling

| N concentration（kg·N·ha^-2^·a^-1^） | Inoculation | 1^st^ days | 5^th^ days | 10^th^ days | 21^st^ days | 30^th^ days |
| --- | --- | --- | --- | --- | --- | --- |
| N0 | Sg | 215.72（6.06）ef | 254.98（7.81）cd | 269.63（6.15）e | 262.28（4.50）c | 265.88（3.76）d |
|  | Pt | 205.71（5.67）f | 231.69（8.93）e | 244.85（3.54）f | 243.63（5.84）d | 240.66（2.26）e |
|  | CK | 191.62（8.43）g | 166.05（8.22）f | 120.41（5.59）g | 113.45（5.81）e | 122.84（4.62）g |
| N30 | Sg | 229.32（3.16）e | 267.79（8.24）c | 291.88（8.16）cd | 285.72（10.36）b | 288.80（7.01）c |
|  | Pt | 242.64（3.31）de | 258.80（7.80）c | 283.84（10.91）d | 286.65（11.24）b | 292.04（5.00）c |
|  | CK | 200.40（13.77）fg | 243.48（4.83）de | 243.31（2.40）f | 236.19（5.41）d | 241.13（4.78）e |
| N60 | Sg | 282.52（13.62）a | 311.57（9.49）a | 322.03（5.06）b | 325.17（7.53）ab | 328.05（5.42）b |
|  | Pt | 287.99（8.55）a | 321.26（7.80）a | 338.45（6.59）a | 335.98（7.01）a | 331.89（3.49）b |
|  | CK | 244.34（10.03）d | 293.88（8.47）b | 295.30（3.30）c | 289.62（8.47）b | 287.32（5.54）c |
| N90 | Sg | 261.58（11.07）bc | 308.23（7.31）a | 332.59（6.50）a | 336.45（5.18）a | 341.89（5.08）a |
|  | Pt | 271.50（7.06）ab | 323.06（4.18）a | 338.20（4.04）a | 334.84（1.97）a | 336.61（7.11）ab |
|  | CK | 247.37（10.86）cd | 282.08（10.25）b | 283.01（6.00）d | 282.48（6.19）b | 288.67（4.03）c |

Note: Vertical bars illustrate standard errors of means(n=3). CK: control. Sg: *Suillus grevillei.* Pt: *Pisolithus tinctorius*. N0: 0 kg·N·hm^-1^a^-1^. N30: normal deposition 30 kg·N·hm^-1^a^-1^. N60: moderate deposition 60 kg·N·hm^-1^a^-1^. N90: severe deposition 90 kg·N·hm^-1^a^-1^. 1^st^ -30^th^ days is the date after pluse-labelling. Numbers show the weighted mean of each treatment. Standard error is shown in brackets(p＜0.05). Different letters indicate significant differences (Differences in treatments were analyzed with the Kruskal-Wallis rank sum test. Significant tests (p < 0.05) were followed by Duncan's test of multiple comparisons).

**Table S3** | The changes of δ^13^C value in branches of the plant under different treatment after pluse-labelling

| N concentration（kg·N·ha^-2^·a^-1^） | Inoculation | 1^st^ days | 5^th^ days | 10^th^ days | 21^st^ days | 30^th^ days |
| --- | --- | --- | --- | --- | --- | --- |
| N0 | Sg | 293.12（3.35）d | 317.34（9.01）ef | 191.17（6.95）f | 199.64（6.43）g | 189.33（6.50）ef |
|  | Pt | 282.59（7.14）de | 322.56（6.96）e | 198.95（6.96）ef | 218.09（6.95）ef | 193.11（6.15）def |
|  | CK | 226.51（9.07）f | 269.52（7.43）g | 115.23（8.04）g | 181.24（7.62）h | 168.18（7.03）g |
| N30 | Sg | 292.13（6.40）d | 372.78（8.85）c | 232.06（10.91）cde | 204.77（6.82）g | 186.76（5.96）f |
|  | Pt | 270.59（6.28）e | 356.55（6.97）d | 240.10（8.82）bc | 233.46（747）cd | 199.15（6.01）cde |
|  | CK | 233.40（7.43）f | 300.18（6.70）f | 197.88（7.82）f | 188.67（3.80）h | 156.37（6.35）h |
| N60 | Sg | 357.01（8.51）a | 395.89（6.94）ab | 254.41（2.21）a | 238.65（4.53）bc | 219.29（2.26）a |
|  | Pt | 348.00（10.87）ab | 402.33（2.12）a | 249.21（6.12）ab | 252.04（4.96）a | 216.23（4.88）a |
|  | CK | 317.84（7.20）c | 375.90（9.82）bc | 235.27（4.30）cd | 217.94（4.88）ef | 201.73（8.36）cd |
| N90 | Sg | 335.68（9.57）b | 375.22（7.33）bc | 238.51（5.51）bc | 227.90（6.97）de | 209.73（7.56）abc |
|  | Pt | 342.03（4.83）b | 388.62（0.60）ab | 223.38（7.02）de | 244.58（2.18）ab | 214.85（5.17）ab |
|  | CK | 283.11（3.82）de | 343.43（5.74）de | 209.44（7.13）e | 215.86（7.01）f | 203.64（7.43）bcd |

Note: Vertical bars illustrate standard errors of means(n=3). CK: control. Sg: *Suillus grevillei.* Pt: *Pisolithus tinctorius*. N0: 0 kg·N·hm^-1^a^-1^. N30: normal deposition 30 kg·N·hm^-1^a^-1^. N60: moderate deposition 60 kg·N·hm^-1^a^-1^. N90: severe deposition 90 kg·N·hm^-1^a^-1^. 1^st^ -30^th^ days is the date after pluse-labelling. Numbers show the weighted mean of each treatment. Standard error is shown in brackets(p＜0.05). Different letters indicate significant differences (Differences in treatments were analyzed with the Kruskal-Wallis rank sum test. Significant tests (p < 0.05) were followed by Duncan's test of multiple comparisons).

**Table S4** | The changes of δ^13^C value in stem of the plant under different treatment after pluse-labelling

| N concentration（kg·N·ha^-2^·a^-1^） | Inoculation | 1^st^ days | 5^th^ days | 10^th^ days | 21^st^ days | 30^th^ days |
| --- | --- | --- | --- | --- | --- | --- |
| N0 | Sg | 207.78（8.21）e | 252.72（3.26）f | 200.72（6.88）g | 184.11（1.98）e | 172.48（10.80）e |
|  | Pt | 225.68（9.12）d | 270.62（2.18）de | 211.69（3.68）f | 205.68（9.26）d | 186.06（3.01）d |
|  | CK | 191.37（8.41）f | 162.34（6.99）g | 115.23（8.04）h | 121.29（5.74）f | 140.30（4.91）f |
| N30 | Sg | 247.85（4.00）c | 274.04（7.99）de | 241.42（6.02）d | 233.58（4.27）c | 226.58（8.16）c |
|  | Pt | 240.15（4.08）c | 285.75（7.19）d | 250.78（2.93）c | 248.01（6.85）b | 240.14（4.49）b |
|  | CK | 204.86（8.80）e | 238.61（7.26）gh | 210.96（5.04）f | 204.27（7.97）d | 195.43（4.08）d |
| N60 | Sg | 272.96（10.97）a | 314.25（8.96）a | 276.30（1.15）a | 273.06（3.03）a | 250.45（11.03）ab |
|  | Pt | 261.57（4.54）ab | 305.22（1.77）ab | 268.84（4.42）ab | 266.35（6.76）a | 250.03（3.40）ab |
|  | CK | 239.08（6.49）c | 263.28（3.36）ef | 251.60（6.24）c | 240.16（6.99）bc | 221.39（3.96）c |
| N90 | Sg | 261.82（6.23）ab | 299.08（7.75）bc | 255.83（5.95）c | 274.18（3.88）a | 259.19（7.86）a |
|  | Pt | 252.83（7.43）bc | 291.13（4.64）cd | 265.91（2.38）b | 266.89（8.39）a | 247.62（5.18）b |
|  | CK | 224.07（8.33）d | 253.11（4.87）f | 229.74（6.85）e | 236.33（5.45）c6 | 227.43（6.03）c |

Note: Vertical bars illustrate standard errors of means(n=3). CK: control. Sg: *Suillus grevillei.* Pt: *Pisolithus tinctorius*. N0: 0 kg·N·hm^-1^a^-1^. N30: normal deposition 30 kg·N·hm^-1^a^-1^. N60: moderate deposition 60 kg·N·hm^-1^a^-1^. N90: severe deposition 90 kg·N·hm^-1^a^-1^. 1^st^ -30^th^ days is the date after pluse-labelling. Numbers show the weighted mean of each treatment. Standard error is shown in brackets(p＜0.05). Different letters indicate significant differences (Differences in treatments were analyzed with the Kruskal-Wallis rank sum test. Significant tests (p < 0.05) were followed by Duncan's test of multiple comparisons).

**Table S5** | The variation of the soil ^13^CO_2_ efflux rate under different treatment after pluse-labelling

| N concentration（kg·N·ha^-2^·a^-1^） | Inoculation | 1^st^ days | 5^th^ days | 10^th^ days | 21^st^ days | 30^th^ days |
| --- | --- | --- | --- | --- | --- | --- |
| N0 | Sg | 1.083（0.025）f | 1.347（0.012）fg | 1.000（0.014）de | 0.802（0.011）g | 0.822（0.025）f |
|  | Pt | 1.171（0.072）d | 1.461（0.012）e | 1.018（0.072）d | 0.901（0.005）ef | 0.797（0.023）g |
|  | CK | 0.901（0.042）g | 0.361（0.013）h | 0.419（0.047）f | 0.092（0.036）h | 0.103（0.039）h |
| N30 | Sg | 1.305（0.025）b | 1.648（0.010）cd | 1.297（0.017）b | 1.258（0.019）bc | 1.214（0.011）bc |
|  | Pt | 1.282（0.048）c | 1.554（0.012）d | 1.237（0.072）bc | 1.201（0.010）c | 1.194（0.031）c |
|  | CK | 1.092（0.024）f | 1.258（0.009）g | 0.923（0.036）e | 0.887（0.024）f | 0.793（0.017）g |
| N60 | Sg | 1.337（0.037）a | 1.834（0.072）b | 1.372（0.019）ab | 1.380（0.016）a | 1.315（0.015）a |
|  | Pt | 1.306（0.060）b | 1.957（0.108）a | 1.424（0.072）a | 1.3012（0.007）ab | 1.299（0.018）a |
|  | CK | 1.175（0.055）d | 1.456（0.012）ef | 1.297（0.032）b | 1.190（0.016）c | 1.198（0.014）c |
| N90 | Sg | 1.317（0.037）ab | 1.616（0.069）c | 1.236（0.008）bc | 1.202（0.011）c | 1.161（0.045）d |
|  | Pt | 1.284（0.058）c | 1.652（0.097）c | 1.193（0.018）c | 1.148（0.024）d | 1.088（0.031）e |
|  | CK | 1.137（0.036）e | 1.375（0.086）f | 1.120（0.019）cd | 1.049（0.022）e | 1.020（0.019）e |

Note: Vertical bars illustrate standard errors of means(n=3). CK: control. Sg: *Suillus grevillei.* Pt: *Pisolithus tinctorius*. N0: 0 kg·N·hm^-1^a^-1^. N30: normal deposition 30 kg·N·hm^-1^a^-1^. N60: moderate deposition 60 kg·N·hm^-1^a^-1^. N90: severe deposition 90 kg·N·hm^-1^a^-1^. 1^st^ -30^th^ days is the date after pluse-labelling. Numbers show the weighted mean of each treatment. Standard error is shown in brackets(p＜0.05). Different letters indicate significant differences (Differences in treatments were analyzed with the Kruskal-Wallis rank sum test. Significant tests (p < 0.05) were followed by Duncan's test of multiple comparisons).

**Table S6** | The variation of the soil ^13^CO_2_ accumulation under different treatment after pluse-labelling

| N concentration（kg·N·ha^-2^·a^-1^） | Inoculation | 1^st^ days | 5^th^ days | 10^th^ days | 21^st^ days | 30^th^ days |
| --- | --- | --- | --- | --- | --- | --- |
| N0 | Sg | 1.186（0.381）f | 2.361（0.091）fg | 4.497（0.179）de | 4.567（0.417）de | 4.617（0.324）d |
|  | Pt | 1.264（0.348）d | 2.584（0.554）e | 4.642（0.476）d | 4.674（0.402）d | 4.695（0.818）d |
|  | CK | 0.568（0.018）g | 1.640（0.899）h | 3.798（0.267）f | 3.864（0.227）g | 3.949（0.319）h |
| N30 | Sg | 1.752（0.015）b | 3.116（0.236）cd | 5.243（0.017）b | 5.178（0.183）e | 5.242（0.435）c |
|  | Pt | 1.717（0.038）c | 3.282（0.707）d | 5.116（0.517）bc | 5.265（0.346）b | 5.222（0.713）c |
|  | CK | 0.746（0.256）g | 1.957（0.549）h | 4.039（0.482）f | 4.172（0.562）f | 4.258（0.475）f |
| N60 | Sg | 2.134（0.044）b | 3.836（0.377）cd | 5.545（0.121）b | 5.649（0.313）a | 5.687（0.629）a |
|  | Pt | 2.057（0.156）c | 3.755（0.716）d | 5.305（0.503）bc | 5.576（0.782）a | 5.616（0.761）a |
|  | CK | 0.905（0.274）g | 2.288（0.388）h | 4.314（0.446）f | 4.394（0.188）e | 4.445（0.432）e |
| N90 | Sg | 1.837（0.177）b | 3.292（0.518）cd | 4.956（0.083）b | 5.183（0.281）c | 5.310（0.312）bc |
|  | Pt | 1.797（0.155）c | 3.124（0.728）d | 5.048（0.757）bc | 5.217（0.642）bc | 5.354（0.681）b |
|  | CK | 0.857（0.194）g | 2.061（0.924）h | 4.134（0.548）f | 4.255（0.575）ef | 4.368（0.482）e |

Note: Vertical bars illustrate standard errors of means(n=3). CK: control. Sg: *Suillus grevillei.* Pt: *Pisolithus tinctorius*. N0: 0 kg·N·hm^-1^a^-1^. N30: normal deposition 30 kg·N·hm^-1^a^-1^. N60: moderate deposition 60 kg·N·hm^-1^a^-1^. N90: severe deposition 90 kg·N·hm^-1^a^-1^. 1^st^ -30^th^ days is the date after pluse-labelling. Numbers show the weighted mean of each treatment. Standard error is shown in brackets(p＜0.05). Different letters indicate significant differences (Differences in treatments were analyzed with the Kruskal-Wallis rank sum test. Significant tests (p < 0.05) were followed by Duncan's test of multiple comparisons).
